# Supplementary material for: Fetal and neonatal echocardiographic analysis of biomechanical alterations for the systemic right ventricle heart
Source: PLoS One. 2024 Sep 19;19(9):e0308645. doi: 10.1371/journal.pone.0308645 (PMC11412552; doi:10.1371/journal.pone.0308645)
Supplement: S1 Table — (DOCX) [file pone.0308645.s002.docx]

## S1 Table

Table 1: List of all abbreviations used in the manuscript.

| Quantity | Abbreviation | Definition |
| --- | --- | --- |
| Cardiac Output | CO | Volume flow rate the heart circulates through the body in one minute |
| Stroke Volume | SV | Blood volume pumped out of the ventricles during systole |
| Peak early diastolic  annular velocity | e’ | Maximum velocity the annular plane that holds the AV valve moves during suction filling phase as the ventricle relaxes |
| Peak late diastolic  annular velocity | a’ | Maximum velocity the annular plane that holds the AV valve moves during atrial contraction filling phase |
| Peak systolic  annular velocity | s’ | Maximum velocity the annular plane that holds the AV valve moves as the ventricle contracts |
| Peak early AV  filling velocity | E | Maximum velocity of blood flowing through the AV valve during suction filling phase as the heart relaxes |
| Peak late AV  filling velocity | E | Maximum velocity of blood flowing through the AV valve during atrial contraction phase |
|  | E/e’ | Ratio for the blood flow velocity to the annular plane velocity during the suction filling phase; correlates to filling pressure |
|  | E/e’ | Ratio for the suction filling blood flow velocity to the atrial contraction blood flow velocity |
| Peak global  longitudinal strain | \|GLS\|_max_ | A percentage change in length of the ventricle from the start of systole to the start of diastole |
| Peak systolic GLS  rate | GLSrs | Maximum rate of change of the ventricle length during systole |
| Peak diastolic GLS  rate | GLSre | Maximum rate of change of the ventricle length during diastole |
| Flow energy loss | FEL | Maximum of total energy dissipated by viscous losses over the ventricular volume during diastole |
| Viscous Strength | VS | Maximum of total rotation over the ventricle volume during diastole |
| Intraventricular pressure difference | ΔP | Difference in pressure between the atrioventricular valve and apex |
|  | Suction ΔP | IVPD at the beginning of diastole that helps initiate filling |
|  | Recovery ΔP | IVPD at peak diastole when the flow stops accelerating |
|  | Ejection ΔP | IVPD at peak systole when blood flow rate is highest along the outflow tracts |
| Minimum pressure location | Min. ΔP Location | Spatial location of minimum pressure inside the ventricle during diastole |
