# Supplementary material for: Fetal and neonatal echocardiographic analysis of biomechanical alterations for the systemic right ventricle heart
Source: PLoS One. 2024 Sep 19;19(9):e0308645. doi: 10.1371/journal.pone.0308645 (PMC11412552; doi:10.1371/journal.pone.0308645)
Supplement: S2 Table — (DOCX) [file pone.0308645.s003.docx]

## S2 Table

Table 2: Complete list of echocardiographic measurements obtained from automated analysis platform

|  |  | HLHS RV (n = 10) | CTRL LV (n = 12) | | CTRL RV (n = 12) | |
| --- | --- | --- | --- | --- | --- | --- |
|  |  | Median (IQR) | Median (IQR) | p-value | Median (IQR) | p-value |
| Stroke Volume | Prenatal | $2.91\left( 2.46,4.30 \right)$ | $1.56\left( 1.33,2.02 \right)$ | **0.001** | $2.47\left( 1.83,3.41 \right)$ | 0.129 |
| (ml) | Postnatal | $3.70\left( 2.86,4.59 \right)$ | $2.83\left( 2.52,3.67 \right)$ | 0.187 | $2.59\left( 1.24,3.44 \right)$ | 0.129 |
|  | p-value | 0.545 | **0.001** |  | 0.863 |  |
| Cardiac Output | Prenatal | $435\left( 377,539 \right)$ | $233\left( 209,299 \right)$ | **0.002** | $341\left( 288,439 \right)$ | 0.166 |
| (ml/min) | Postnatal | $539\left( 398,610 \right)$ | $424\left( 265,583 \right)$ | 0.235 | $327\left( 159,471 \right)$ | 0.075 |
|  | p-value | 0.650 | **0.024** |  | 0.603 |  |
| s’ | Prenatal | $2.73\left( 2.10,3.59 \right)$ | $2.70\left( 2.01,3.55 \right)$ | 0.843 | $3.30\left( 1.99,4.29 \right)$ | 0.391 |
| (cm/s) | Postnatal | $2.92\left( 2.32,4.09 \right)$ | $2.45\left( 1.74,2.67 \right)$ | 0.065 | $2.98\left( 2.47,3.38 \right)$ | 0.742 |
|  | p-value | 0.290 | 0.204 |  | 0.686 |  |
| e’ | Prenatal | $2.85\left( 2.44,4.45 \right)$ | $3.50\left( 3.03,4.15 \right)$ | 0.644 | $4.33\left( 3.45,5.49 \right)$ | 0.129 |
| (cm/s) | Postnatal | $4.03(349,5.22)$ | $2.98\left( 2.55,3.85 \right)$ | 0.138 | $3.52\left( 2.75,4.64 \right)$ | 0.510 |
|  | p-value | 0.290 | 0.564 |  | 0.326 |  |
| a’ | Prenatal | $2.00\left( 1.00,3.42 \right)$ | $2.40\left( 1.55,3.68 \right)$ | 0.568 | $3.25\left( 1.25,5.07 \right)$ | 0.157 |
| (cm/s) | Postnatal | $3.29\left( 2.05,4.18 \right)$ | $1.28\left( 0.64,2.01 \right)$ | **0.013** | $2.00\left( 1.74,2.50 \right)$ | **0.045** |
|  | p-value | 0.180 | **0.032** |  | 0.063 |  |
| E | Prenatal | $44.8(34.4,48.0$) | $29.7\left( 28.2,34.5 \right)$ | **0.027** | $37.3\left( 35.0,38.9 \right)$ | 0.114 |
| (cm/s) | Postnatal | $62.1\left( 60.6,68.0 \right)$ | $41.1\left( 36.5,45.8 \right)$ | **0.003** | $34.9\left( 30.5,43.3 \right)$ | **<0.001** |
|  | p-value | **0.001** | **0.030** |  | 0.436 |  |
| A | Prenatal | $33.2\left( 31.5,37.2 \right)$ | $20.8\left( 18.1,28.1 \right)$ | **0.012** | $23.5\left( 12.6,33.3 \right)$ | **0.048** |
| (cm/s) | Postnatal | $37.4\left( 33.9,54.1 \right)$ | $31.6\left( 29.5,35.6 \right)$ | 0.121 | $30.1\left( 23.6,35.2 \right)$ | **0.035** |
|  | p-value | 0.082 | **0.005** |  | 0.453 |  |
| E/e’ | Prenatal | $14.8\left( 7.1,23.3 \right)$ | $8.6\left( 7.6,11.2 \right)$ | 0.210 | $9.9(5.7,11.6$ | 0.129 |
|  | Postnatal | $15.4\left( 13.3,17.9 \right)$ | $13.2\left( 7.7,16.8 \right)$ | 0.262 | $9.9\left( 7.9,11.0 \right)$ | **0.004** |
|  | p-value | 0.762 | 0.312 |  | 0.773 |  |
| E/A | Prenatal | $1.26\left( 1.14,1.37 \right)$ | $1.32\left( 1.25,1.47 \right)$ | 0.468 | $1.40\left( 1.16,3.11 \right)$ | 0.210 |
|  | Postnatal | $1.41\left( 1.16,1.82 \right)$ | $1.16\left( 0.92,1.44 \right)$ | 0.086 | $1.16\left( 0.89,2.23 \right)$ | 0.235 |
|  | p-value | 0.272 | 0.141 |  | 0.174 |  |
| \|GLS\|_max_ | Prenatal | $18.7\left( 15.1,25.1 \right)$ | $15.8\left( 13.5,17.0 \right)$ | 0.138 | $17.8\left( 16.2,18.8 \right)$ | 0.843 |
| (%) | Postnatal | $16.9\left( 15.4,19.2 \right)$ | $17.9\left( 16.8,20.7 \right)$ | 0.235 | $23.4\left( 19.5,25.8 \right)$ | **0.008** |
|  | p-value | 0.427 | **0.020** |  | **0.011** |  |
| GLSrs | Prenatal | $1.38\left( 1.25,1.60 \right)$ | $2.14\left( 1.89,2.27 \right)$ | **0.010** | $1.75\left( 1.56,2.15 \right)$ | **0.005** |
| (s^-1^) | Postnatal | $1.43\left( 1.04,1.54 \right)$ | $1.42\left( 1.27,1.66 \right)$ | 0.306 | $1.81\left( 1.66,2.21 \right)$ | **0.004** |
|  | p-value | 0.650 | **0.024** |  | 0.908 |  |
| GLSre | Prenatal | $1.28\left( 0.99,2.26 \right)$ | $1.11\left( 0.90,1.50 \right)$ | 0.448 | $1.34\left( 1.16,1.48 \right)$ | 0.947 |
| (s^-1^) | Postnatal | $0.83\left( 0.51,1.17 \right)$ | $1.57\left( 1.02,1.98 \right)$ | **0.025** | $1.58\left( 1.11,2.23 \right)$ | **0.035** |
|  | p-value | **0.045** | 0.260 |  | 0.248 |  |
| Flow energy loss | Prenatal | $23.2\left( 11.2,36.3 \right)$ | $4.9\left( 3.1,6.1 \right)$ | **0.003** | $8.3\left( 5.9,10.4 \right)$ | **0.048** |
| (mW/m) | Postnatal | $31.8\left( 23.5,39.9 \right)$ | $10.8\left( 6.5,15.5 \right)$ | **0.002** | $8.2\left( 6.1,9.1 \right)$ | **<0.001** |
|  | p-value | 0.199 | **0.013** |  | 0.686 |  |
| Kinetic Energy | Prenatal | $27.5\left( 21.9,29.8 \right)$ | $4.2\left( 3.6,5.1 \right)$ | **<0.001** | $8.2\left( 7.0,10.6 \right)$ | **<0.001** |
| (mJ/m) | Postnatal | $25.7\left( 19.3,33.2 \right)$ | $10.8\left( 4.0,13.8 \right)$ | **0.002** | $5.0\left( 4.4,6.1 \right)$ | **<0.001** |
|  | p-value | 0.880 | **0.050** |  | **0.017** |  |
| Vortex Strength | Prenatal | $248\left( 197,326 \right)$ | $97\left( 77,102 \right)$ | **<0.001** | $135\left( 123,145 \right)$ | **0.001** |
| (cm^2^/s) | Postnatal | $315\left( 258,342 \right)$ | $180\left( 121,263 \right)$ | **0.005** | $132\left( 98,150 \right)$ | **<0.001** |
|  | p-value | 0.140 | **0.004** |  | 0.686 |  |
| Suction ΔP | Prenatal | $0.31\left( 0.19,0.36 \right)$ | $0.24\left( 0.21,0.30 \right)$ | 0.229 | $0.18\left( 0.07,0.19 \right)$ | **0.034** |
| (mmHg) | Postnatal | $1.27\left( 0.81,1.46 \right)$ | $0.54\left( 0.33,0.64 \right)$ | **0.001** | $0.67\left( 0.41,0.72 \right)$ | **0.006** |
|  | p-value | **<0.001** | **<0.001** |  | **<0.001** |  |
| Recovery ΔP | Prenatal | $-1.43\left( \begin{aligned} -1.96, \\ -0.89 \end{aligned} \right)$ | $-0.42\left( \begin{aligned} -0.50, \\ -0.34 \end{aligned} \right)$ | **<0.001** | $-0.55\left( \begin{aligned} -0.62, \\ -0.47 \end{aligned} \right)$ | **0.002** |
| (mmHg) | Postnatal | $-2.26\left( \begin{aligned} -2.46, \\ -1.97 \end{aligned} \right)$ | $-0.73\left( \begin{aligned} -1.06, \\ -0.49 \end{aligned} \right)$ | **0.002** | $-0.38\left( \begin{aligned} -0.68, \\ -0.31 \end{aligned} \right)$ | **<0.001** |
|  | p-value | **0.007** | **0.005** |  | 0.184 |  |
| Ejection ΔP | Prenatal | $0.12\left( 0.04,0.28 \right)$ | $0.15\left( 0.10,0.28 \right)$ | 0.704 | $0.09\left( 0.03,0.14 \right)$ | 0.585 |
| (mmHg) | Postnatal | $0.43\left( 0.15,0.67 \right)$ | $0.26\left( 0.11,0.84 \right)$ | 0.957 | $0.12\left( 0.03,0.39 \right)$ | 0.108 |
|  | p-value | 0.061 | 0.098 |  | 0.113 |  |
| Min. ΔP Loc. | Prenatal | $9.2\left( 7.5,10.0 \right)$ | $4.1\left( 3.8,4.4 \right)$ | **0.001** | $4.8\left( 3.9,5.3 \right)$ | **0.004** |
| (mm) | Postnatal | $4.9\left( 4.3,5.7 \right)$ | $4.4\left( 2.8,5.7 \right)$ | 0.428 | $2.9\left( 2.3,3.5 \right)$ | **0.007** |
|  | p-value | **0.003** | 0.862 |  | **0.028** |  |
