# Supplementary material for: Fetal and neonatal echocardiographic analysis of biomechanical alterations for the systemic right ventricle heart
Source: PLoS One. 2024 Sep 19;19(9):e0308645. doi: 10.1371/journal.pone.0308645 (PMC11412552; doi:10.1371/journal.pone.0308645)
Supplement: Attachment — Submitted filename: Response for Reviewers.docx [file pone.0308645.s005.docx]

## Overview and response structure

We thank the reviewers for their valuable input throughout the review process. We have addressed their revisions to clarify the presented work further. Each point is addressed with a response and the corresponding excerpt from the revised final manuscript. Our response is provided in red Arial, and the text excerpt is in blue Arial.

## Response to reviewers

**Reviewer #1:**

I congratulate with authors for their novel type of echocardiography examination.

The manuscript is well written in each section and well-structured with appropriate attention for style!

As an Obstetrician involved in prenatal ultrasound diagnosis, I don’t have the appropriate background to evaluate the mathematics calculation behind the formulas. Images and Table are of high quality.

Thank you for the strong support of our work.

**Reviewer #2:**

Thank you for providing the opportunity to review the paper titled 'Fetal and Neonatal Echocardiographic Analysis of Biomechanical Alterations for the Systemic Right Ventricle Heart.' In this paper, the authors reported on changes in diastolic function of the SRV using a novel ultrasonographic method during both the prenatal and postnatal periods. The manuscript was well-written, and I have no concerns regarding this paper.

Thank you for the strong support of our work.

**Reviewer #3:**

This is a small study exploring the biomechanics of the single right ventricle during perinatal transition, particularly focusing on novel measures.

In my opinion, this study needs to be reframed as a technical paper. The study is too small and the variety of patient anatomy too great to “understand the fetal and neonatal SRV” as stated in the abstract.

Thank you for your thorough feedback on our work. We hope to address each of your points in a manner that helps reframe the work sufficiently.

Abstract

The background could better reflect the study. The objective of the study seems to be to compare standard echo measures to novel measures using a range of SRV case types. In my opinion, the study is largely a technical paper about use of novel biomechanical measures in a small group of patients.

The aims of this paper have been adjusted to focus the background on elucidating changes across the perinatal transition for SRVs and health bi-ventricle hearts. The amended text for the Background and Objectives of the Abstract is provided below.

**Background** The perinatal transition's impact on systemic right ventricle (SRV) cardiac hemodynamics is not fully understood. Standard clinical image analysis tools fall short of capturing comprehensive diastolic and systolic measures of these hemodynamics.

**Objectives** Compare standard and novel hemodynamic echocardiogram (echo) parameters to quantify perinatal changes in SRV and healthy controls.

Introduction

The introduction lacks cohesion and after reading it, I was still not clear what this study was about. It does not address a) perinatal transition or b) neonatal hemodynamics of the SRV. I would encourage the authors to clarify what the study is really about: that hemodynamic changes in the SRV during perinatal transition are poorly understood, and that current measures may insufficiently capture systolic and diastolic function. Then, the aims of the study are to compare novel and standard measures to better describe perinatal changes.

We acknowledge the points the reviewer has made. We have revised this section to better tie the background and motivation to the manuscript objectives. The amended text is provided below.

The perinatal transition induces significant hemodynamic changes in newborns, involving four major events: loss of umbilical cord blood flow, first breath, circulatory shift, and myocardial performance changes [1]. Subsequently, the lungs become the primary respiratory organ, necessitating fluid clearance, leading to decreased systemic venous return and increased systemic resistance [2]. Left ventricle (LV) and right ventricle (RV) hemodynamics show a marked increase in cardiac output (CO) – LV output rises by 300%, and RV output by 150% [3], resulting from alterations in preload, afterload, and pericardial pressure effects.

In both SRV and normal bi-ventricles, changes in pericardial pressure affect ventricular expansion during diastolic filling, influencing blood flow patterns, also known as flow fields. Current guidelines primarily focus on capturing systolic function measurements, e.g., stroke volume, fractional area change, tricuspid annular plane systolic excursion, or tissue Doppler-derived peak systolic annular velocity (s’). However, these measurements are often inaccurate, highly variable, and influenced by factors such as image quality, probe placement, heart size, and tissue motion [4,5]. Consequently, there is a lack of clinical understanding regarding the assessment of diastolic function in the RV, which undergoes significant alterations in the perinatal period and is crucial for SRV physiology [6,7].

Little is known regarding these flow fields and their effects on cardiac hemodynamics in both prenatal and postnatal states. Initial work employed 4D flow MRI to examine cardiac chamber flow in newborns [8]. While bulk flow features were reliably observed, finer features typical in these flows remained unresolved. Subsequent studies on congenital heart defects (CHDs) using 4D flow MRI are scarce [9] but no additional studies of this kind exist. However, the evolving potential of the fetal 4D flow MRI [10,11], offering higher resolution voxel sizes, may address this gap. Still, it is difficult to perform fetal 4D flow MRI due to uncontrollable factors including fetal motion, breathing, and positioning of the mother and fetus. Recently, echocardiography-based methods explored cardiac flow in fetal and neonate patients, utilizing a blood speckle imaging [12] or color Doppler flow reconstruction [13]. Although demonstrated for feasibility, none have explored their potential for understanding diastolic function across the perinatal period. Quantitative tools can resolve flow-induced vortices, energy losses, and pressure distributions, aiding in characterizing abnormal flow patterns in SRV hearts [14].We have recently carried out a similar study in tetralogy of Fallot patients [15].

In the current study, we applied an integrated and automated echocardiography analysis method for measuring cardiac biomechanics from fetal and neonatal echocardiograms to quantify standard and novel hemodynamics biomarkers. Our goal was to apply novel, integrated echo-based imaging tools to better understand the hemodynamic changes in the SRV during the transition from fetal to postnatal life. The analysis collected chamber, annular motion, strain, and hydrodynamics parameters. The tools employed are vendor-agnostic, and do not rely on heuristics adopted from adult echocardiography. These advancements enable conventional and novel biomechanics measurements to be robustly collected from fetal and neonatal echocardiograms.

Our study explored how biomechanics parameters quantified from echocardiography change from late gestation (> 28 weeks gestational age) and day of birth for SRVs and the healthy left (LV) and RV. Here, we compared standard and novel echo-based approaches to better understand the perinatal changes in the SRV and normal heart. We hypothesize that the SRV has altered diastolic flow compared to both ventricles of the healthy heart, captured by the biomechanics parameters. The SRV presents a challenging clinical scenario that emphasizes the need for robust parameters to understand patient cardiac health comprehensively.

Line 66 – 70 discuss benefits of fetal detection and that pediatric echo is used to treat. Whilst true, these statements aren’t really relevant to the paper.

We have addressed this in a response to the reviewer’s previous comment.

Line 71 – remove “i.e.”. SV and CO are not the only measures of systolic function and are not routinely reported in most centres. It would be better to say “e.g.” and quote measures more commonly used, such as RVFAC, TAPSE, TDI S’.

We have addressed this in a response to the reviewer’s previous comment.

Line 77 – what do the authors mean by “hemodynamics” here? This is too vague; I am genuinely uncertain what they are referring to, and the reference is to an overall guideline from 2004. As an aside, there are several more recent AHA / JASE guidelines on Fetal Echo – the Donofrio 2014 one is quoted. Moon-Grady 2023 should also be quoted: <https://doi.org/10.1016/j.echo.2023.04.014>.

We have attempted to clarify the use of *hemodynamics*. Previously, we were using hemodynamics to include the *hydrodynamics* or *parameters related to the flow of blood*. We have thus tried to use *hemodynamics* to describe the flow of blood through the cardiovascular system while using *hydrodynamics* to relate to the physics – motion, pressure, energy loss, etc. – that manifest due to blood flow.

We have also addressed the aside by updating our reference from the Donofrio 2014 guideline to the Moon-Grady 2023 guideline.

Line 79 – 82: I think this paragraph needs rewording. Software does not rely on automated segmentation to work, as the workflow is to manually trace the chambers. Perhaps “rely” is not the correct word? The discussion regarding segmentation is unclear, and not well referenced – only to overall guidelines. Do they mean segmentation of the image into an LV / RV for example? I think this is what they mean, and if better defined, it will help to clarify the other points. This is indeed manual. Or segmentation of the ventricles? This is done automatically by software (Tomtec, EchoPac, Syngo). Strain is not difficult to obtain, for example, a global longitudinal strain measurement on a fetal study. Strain does not “rely” on ventricular segmentation to my knowledge, although of course it relies on segmentation of the image (identification of the ventricle). This is a somewhat superficial point in a paper which is not really about automatically finding parts of the image (e.g. through an AI algorithm). The authors approach also relies upon manual identification of the annulus and apex. It is true that dividing the ventricle, particularly a SRV, into, for example, a six-segment model, is difficult, and whether the septum should be included or not (as it constitutes a variable amount of wall) in a HLHS fetus / infant is unclear.

We have addressed this in response to the reviewer’s previous comment.

Methods

The methodology is largely a detailed mathematical description of analysis. This is beyond my ability to review critically.

We appreciate your candidness. The mathematical foundation is important to this work. Thus we included all of our mathematical descriptions to add transparency to the work, over having interested readers open referencing articles as needed to understand how our work was performed.

It is unclear what frame rates the images were taken at or stored at. Given the rest of the analysis is likely frame-rate dependent, and the high heart rates in fetuses and neonates, this should be quoted.

It is reasonable to assume that reporting frame rates would enhance overall understanding of the work. Frame rate varied between the fetus and neonate time points, mainly due to imaging depths. This quantity is reported as median and (IQR); as follows, for fetal imaging, the frame rate was 18 (15,24) Hz for controls and 17 (17,22) Hz for SRV, and for neonatal imaging, the frame rate was 27 (18,30) Hz for controls and 29 (19,40) Hz for SRV. We have amended the manuscript with the following text to report these quantities.

Frame rates varied between the prenatal and postnatal sessions due largely to differences in imaging depths. The median, maximum, and minimum values are reported in Table 1.

*Table 1: Frame rates for apical four-chamber scans within the cohort*

|  |  | B-mode Echocardiography | | | Color Doppler Echocardiography | | |
| --- | --- | --- | --- | --- | --- | --- | --- |
| Frame rate (FPS) | | Minimum | Median | Maximum | Minimum | Median | Maximum |
| SRV | Prenatal | 22 | 77 | 85 | 17 | 21 | 62 |
|  | Postnatal | 40 | 76 | 101 | 19 | 29 | 40 |
| CTRL | Prenatal | 42 | 66 | 87 | 15 | 18 | 24 |
|  | Postnatal | 30 | 30 | 95 | 18 | 27 | 30 |

Line 112: I believe the machine was an SC2000 – there is a zero missing.

The text has been corrected to provide the correct machine name.

The authors state that automated speckle tracking of the mitral annulus has been previously validated. Indeed, speckle tracking includes the annulus in general. However, as the authors spell out at length the process for tracking the annulus, is the algorithm new in some way? If so, how has it been validated? If not different from standard speckle tracking, what is new?

We explained the annulus tracking process in detail to clarify that our approach is Fourier domain-based (spectral). Our methodology implementation does not differ from past approaches, we provided this explanation for transparency to ensure the reader comprehends the algorithm used for annulus tracking.

The peak annulus velocities in standard TDI differ from this process, as no tracking is performed – it is derived from movement of the annulus THROUGH a static pulse-wave sector. Therefore, the normal values are likely different. Is there prior data defining a normal range in fetuses and newborns, and how it compares to standard TDI? The reference [21] refer to accuracy using adult validation – much lower heart rates. You cannot extrapolate.

We recognize that reference [21] pertains to adult peak annulus measurements, and these values cannot be extrapolated to fetal and neonate data due to differences in frame rates and discrete pixel size of speckle patterns. This study is the first to implement this methodology for fetal and neonates. However, signal quality, crucial for algorithm performance, is affected by factors like heart rate and adipose tissue in adults, whereas fetal and neonate cases may offer more ideal conditions for this method under appropriate settings.

We have edited a portion of the methods section to indicate that this approach is validated with adult patients.

Automated speckle tracking mitral annulus position and velocity measurements has been previously validated with adult patients [21].

Furthermore, we have added text to the Discussion section which mention the limitation of frame rates and the fact this method must undergo further validation against TDI measurements to understand method limitations and where ranges of normal measurement values may fall for fetal and neonatal imaging.

One such instance is the algorithm used for annulus tracking to collect peak tissue motion measurements (s’, e’, a’), where frame rates can impact the ability to resolve measurements that match tissue doppler imaging (TDI).

The reference [25, 2020] for vortices seems to be theoretical and small animal models. It there a follow-up in humans? What would be considered the gold standard? It seems that the authors have moved strait to assessing pathology using this method.

We recently published a study in JASE using the DoVeR method to reconstruct flows in tetralogy of Fallot patients entering young adulthood, which we reference in the introduction (reference [17]). In addition, a separate manuscript is under review in which we show work comparing DoVeR and 4D flow MRI (article title and manuscript number provided below). Our results show a high correlation between methods, suggesting that DoVeR can provide comparable flow parameter measurements, including vortex strength, to 4D flow MRI.

*Meyers, B.A., Zhang, J., Nyce, J., Loke, Y.H., and Vlachos, P.P. “Enhanced echocardiographic assessment of intracardiac flow in congenital heart disease.” Under review with PLOS One. Manuscript Number: PONE-D-23-30513R1.*

Line 149: what limitations are the authors referring to – this is not clear.

We acknowledge this statement was unclear and seemingly not tied to prior examples. The text has been amended to clarify what limitations to strain estimation we are referring.

A novel algorithm is used to measure global longitudinal strain (GLS) from the whole ventricle image [22]. This algorithm overcomes limitations for strain estimation associated with manual tracings of the chambers [4,14] and the use of speckle pattern matching kernels [23,24] which are sensitive to image spatial and temporal resolution.

Line 151: GLSr is not defined at first use, and not a standard abbreviation.

Thank you for noting this error. The text has been corrected appropriately.

Line 184: There is a sentence fragment.

The text has been corrected appropriately.

Line 188: Segmentation using Simpson’s rule – do the authors mean calculation of volume based on length and cross-sectional ellipses? Perhaps this needs to referenced (if one exists) as accurate for a single right ventricle, which is not conical?

In our study, we employ Simpson’s rule – or "method of disks" – for volume calculation as it is commonly defined, by multiplying the ventricle's length with the sum of ellipsoid disk cross-sectional areas. While accuracy for the single right ventricle is not directly documented, past research shows Simpson’s rule's reliability for biventricular fetal scans (reference provided below). As significant remodeling is unlikely in the perinatal period, similar findings may be applicable. For the single right ventricle, Simpson’s rule has been applied clinically, although precise volume measurements are challenging. Reporting volumes aligns with current practices, but acknowledging this limitation is essential (reference provided below).

*Schmidt, K.G., Silverman, N.H., Van Hare, G.F., Hawkins, J.A., Cloez, J.L. and Rudolph, A.M., 1990. Two-dimensional echocardiographic determination of ventricular volumes in the fetal heart. Validation studies in fetal lambs. Circulation, 81(1), pp.325-333.*

*Silverman, N.H. and McElhinney, D.B., 1998. Echocardiography of hypoplastic ventricles. The Annals of thoracic surgery, 66(2), pp.627-633.*

We have amended the text to include the necessary references to support the accuracy of Simpson’s rule in measuring ventricular volumes in fetal echocardiograms.

Previous research demonstrated the accuracy of Simpson's rule in estimating actual volume using animal models in fetal echocardiography [27].

Additionally, in the limitations portion of the Discussion, we have noted the limitations of this technique as well as included the relevant reference to support this claim.

Another instance is the use of the Simpson rule to compute volumes for the SRV hearts, which can be abnormally shaped. While volume estimates may hold clinical value, they may not represent the true volume, introducing greater uncertainty into subsequent calculations, such as ventricular mass [34].

No explanation on how the SV / CO, or many of the other standard echo measures were obtained is reported. These can be done using geometric assumptions or the continuity equation. This should be specified.

We recognize the need for additional details on how each reported measure was obtained. Relevant details have been included within the manuscript where appropriate. An excerpt clarifying the measurement of SV and CO is now provided.

End-diastolic volume (EDV) was measured at the initial volume of each cycle in the processed volume timeseries. End-systolic volume (ESV) was determined using peak finding for the minimum volume in the cardiac cycle. Stroke volume (SV), calculated as the difference between EDV and ESV for each cycle, and cardiac output (CO), representing the stroke volume multiplied by heart rate, serve as output measures for systolic function.

Results

Restrictive patent foramen ovale is not a subtype of SRV – this needs clarification. Surely, as the authors have the images, the subtype should be able to be reported on all the studies – one is not.

Two of the patients have L-looped ventricles, one cardiogenetic shock as a complication, one total anomalous veins and one moderate TR! This is a highly heterogenous group hemodynamically. I don’t think you can put them all together and draw any conclusions about the SRV. This loops back to the paper being largely technical – the differences in data that are obtained from the novel methodologies may be useful, even in a heterogenous group. If the authors disagree, further anatomical description / explanation to justify this is required.

We acknowledge the diversity within our cohort, which encompasses a range of anatomical features that may limit the significance of clinical or biomechanical findings. However, in response to the reviewer's feedback, we have transitioned our work towards a more technical focus. Consequently, our demonstration across a diverse group of patients with variable volume loading showcases the capability of the tools employed to evaluate a wider spectrum of conditions. We maintain the inclusion of these individuals in the analysis for the present study.

Nevertheless, it is imperative to emphasize to readers that the small, highly heterogeneous nature of our cohort precludes drawing conclusions that extend to the broader population of SRV patients. Accordingly, we have revised the text to underscore this point.

Moreover, the cohort comprises a diverse population of SRV subjects exhibiting varying hemodynamics and volume loading conditions. Given this diversity, drawing definitive conclusions regarding altered biomechanics and hemodynamics during the perinatal transition in SRV patients necessitates investigation in cohorts with stringent exclusion criteria, focusing on individuals with more consistently similar findings.

In reporting, more clarity on which results (fetal vs newborn) are being reported is needed.

Proper labels for several subsections in the Results were not clearly provided to indicate which were associated with fetal and which with newborns. The text has been amended accordingly.

The authors reference and interpret diastolic parameters using guidelines which, to my knowledge, are not validated, and have evidence that they do not necessarily reflect dysfunction in either fetuses, neonates or SRV. I recommend that they refrain from interpretation, and just describe the parameters they evaluated. Ref 20 is not the most recent and is focussed on adults.

We recognize that the referenced guidelines and interpretation may not align with our population's characteristics. Although our original intent was to offer insights for non-expert readers regarding parameters associated with clinical findings, we acknowledge that this approach could be misleading. As a result, any reference to [20] in the discussion of diastolic parameters has been removed.

Do the authors have any data on correlation between measures such as IVPD and E/e’?

Given this study is retrospective and the cohort size limitation, we have not extensively explored associations between parameters. However, recognizing the value of comparing early filling (suction) IVPD with E/e’, a parameter associated with filling pressure in adults (with certain assumptions), we aim to explore this further in future works with more rigorously implemented study protocols.

Discussion

Lines 292 – 319 is largely data that is already known about the SRV using standard echo techniques and could be more efficiently discussed.

The amended text is provided.

Assessing diastolic function non-invasively in perinatal SRV patients remains challenging. Standard measurements from echo poorly correlate with diastolic function, especially in the perinatal transition [31], precluding the development of well-timed primary prevention strategies. This study explored the use of fetal and neonatal echo to quantify cardiac biomechanics, comparing standard and novel parameters of cardiac function for better understanding of perinatal changes in normal and SRV hearts. The work further demonstrates that novel hydrodynamic parameters, which can be quantified from scans collected during anatomy ultrasounds, can also detect functional changes in the SRV.

In SRV defects, the RV undergoes remodeling to support pulmonary and systemic circulations, reflected by quantified standard hemodynamic parameters using echo – SV and CO. SRV patients consistently exhibited larger SV (2.91 mL *in utero*, 3.70 mL *ex utero*) compared to the control RV (2.47 mL *in utero*, 2.59 mL *ex utero*) and the control LV (1.56 mL *in utero*, 2.84 mL *ex utero*). Similar trends were observed in CO, a function of SV and heart rate. This suggests that SRV enlargement is a response to meet blood volume requirements and begins during the fetal timeframe.

During the perinatal transition, standard diastolic function measurements showed little change. However, consistent significant differences were observed between SRV and control patients. SRV E/e’ was consistently higher (17.5 *in utero*, 15.7 *ex utero*) than the control RV (8.8 *in utero*, 10.1 *ex utero*) and the control LV (8.5 *in utero*, 11.4 *ex utero*). This may also be associated with elevated IVPD required to empty and fill the SRV (Fig. 1e-2, Fig. 2e-2). Together, the SRV may require elevated filling pressures to overcome increased ventricular stiffness and reduced contractility.

The early and late diastole phases were consistently fused in SRV subjects (as illustrated in Fig. 1e-1, Fig. 2e-1). Prenatally fusion stems from the SRV needing to meet CO demands, which is affected by preload and afterload affects. Postnatally this altered filling pattern becomes more apparent with the onset of spontaneous respirations and the predicted fall in pulmonary vascular resistance.

The increased SRV volume allows for altered diastolic flow, exhibited in larger vortex formations (Fig. 1c, Fig. 2c), more disorganized flow (Fig. 1d, Fig. 2d), and greater flow energy loss (Fig. 1d and Fig. 2d). In healthy hearts, a donut-like vortex ring forms at the annular valve leaflet tips [32], which appears as two counter-rotating vortexes, as seen in Fig. 3. Normally, these vortices help aid in efficient ventricular filling [33]. In the SRV, the free wall vortex occupies a larger area because of the increased volume, resulting in greater flow energy loss which reduces efficient flow redirection prior to systolic ejection. These observations are consistent with our recent study in tetralogy of Fallot patients [15].

The discussion of diastolic function is logically flawed – the authors first note that “conventional imaging measures do not correlate with diastolic function”, but then use the same measures to make conclusions, for example, that the filling pressures are elevated. The E/e’ in a fetus / neonate with altered filling conditions and afterload may not mean the same thing as in an adult or even a normal neonate. Again, the reference is to a document focussed on adults. And not for the right ventricle. The evidence quoted from Line 328 is fairly weak – the MPI is not a strong measure of DD. More reliance on atrial contraction can be due to higher volume requirements. Without a measure of wall stress / filling pressures, the impact on the ventricular muscle in terms of pathways leading to fibrosis are unknown.

Addressed by the amended text provided in a preceding response. We have deleted references to adult studies and focused only on the perinatal time.

Line 315 319: The phases of inflow are explained as due to exposure to systemic and pulmonary pressure. This is likely not the reason and requires clarification. The SRV in utero is exposed to higher diastolic volume loading and required to produce higher cardiac output. The preload will depend on the compliance of the ventricle (it may be able to maintain low EDP), and the afterload on systolic function, placental function and SVR. The pulmonary pressures should not influence these parameters, and should not influence the relative phases of filling. Filling further increases after birth with falling PVR, so 318 – 319 is likely true.

Addressed by the amended text provided in a preceding response.

Line 340 - Should this be in the limitations section? While it’s unclear what the “above methods” refers to, if the authors refer to the methods they used in this study, I agree that they are not directly applicable without better validation. These techniques need direct correlations with gold standard measures such as invasive animal models or cath lab Ees measures of function before we can draw conclusions. If this data in fact exists, this needs to be elucidated in the discussion and methods.

We acknowledge the reviewer's suggestion to place this sentence in the Study Limitations section, and we have made the correction. We appreciate the reviewer's feedback and want to indicate that we plan to conduct a similar study to quantify parameters in this paper and compare them with invasive catheterization measurements in SRV patients. Our study will compare parameters with pressure-volume loop measurements, encompassing end-diastolic pressure-volume relationships (EDPVR), end-systolic PVR, and end-systolic elastance (Ees), aligning with the reviewer's suggestion.

Limitations: The study is retrospective, with small numbers. The fetal studies were quite early (33 weeks), which means they may not be representative of biomechanics immediately prior to birth.

A sentence to acknowledge this limitation has been included. The amended text is provided below.

Furthermore, the study observed fetuses at 33-weeks gestational age, so the biomechanics quantified may not represent those immediately prior to birth.

**Reviewer #4:**

Hypoplastic Left Heart" uses a novel imaging algorithm to quantify measurements of fetal and neonatal echocardiography in healthy and SRV hearts. The data show not only early diastolic dysfunction in SRV hears compared to healthy controls, but also some subtle differences in systolic function during the neonatal period.

Thank you for your thorough feedback on our work. We hope to address each of your points in a manner that helps strengthen this work sufficiently.

While the image analysis is intricately detailed in the methods, this imaging algorithm to my knowledge is an n=1. One question that I have, is the imaging algorithm used in Matlab for this analysis available on github or some other public forum for other users. This is important as the measurements detailed herein would only be acquired by the sites involved in this particular study and would not be generalizable to a broader user, in particular neonatologists and pediatric cardiologists in a clinical setting.

The algorithms used in this work that are in-house developed are patented. As such, no public hosting on Github or another public forum is being done. The algorithms can be made available to any reader by request after proper licensing agreements are in place.

One of the major concerns in this manuscript is that the data acquired from this algorithm is not compared to any other clinical measurement for the purpose of validation and there is no way to assess whether these measurements are of clinical quality or not. Given that this was a retrospective study, it would seem reasonable that these echoes would have been quantified by health care practitioners and would likely have this data stored in the electronic medical records as a way to compare the algorithm measures of standard measurements such as E, A, E/e', etc.. as a way to compare those standard measurements against the algorithm. Without some way of comparing the algorithm to a gold standard we are only left to assume these measurements are valid. If possible, can the authors provide some comparisons between standard software derived measures against the algorithm.

The reviewer has brought forward an excellent point, that without comparison to complimentary measurement approaches that are considered standard, additional validation is required. In previous key publications, we compared our algorithm measurements for morphology and strain against standard approaches on pediatric and adult echocardiography data.

*Meyers BA, Brindise MC, Kutty S, Vlachos PP. A method for direct estimation of left ventricular global longitudinal strain rate from echocardiograms. Sci Rep. 2022;12: 1–11.*

*Brindise M, Meyers B, Kutty S, Vlachos P. Automated peak prominence-based iterative Dijkstras algorithm for segmentation of B-mode echocardiograms. IEEE Trans Biomed Eng. 2021;69: 1595–1607.*

For measurements such as E and A, our previous works do not compare these measurements. However, the DoVeR method is known to be as accurate than other 2D vector flow mapping (VFM) methods. While we are not aware of any studies with VFM that verify color Doppler derived E or A to pulse wave measurements, it is known that color Doppler measurements slightly underestimate peak values (<10% difference).

*Stewart, S.F., 2001. Effects of transducer, velocity, Doppler angle, and instrument settings on the accuracy of color Doppler ultrasound. Ultrasound in medicine & biology, 27(4), pp.551-564.*

Also, from the statistical analysis the authors used a students t-test to compare differences between groups in this analysis. Since you used a parametric analysis, why weren't the reported values as mean (SD)? Was your data normally distributed and did the authors perform a normality test prior to the use of a parametric test. The authors reported median (IQR) which is fine but usually these values are reported when using non-parametric tests like the Wilcoxon.

This was an error on our part based on the method used. We should not have used the Student’s t-test because (1) we did not report means and standard deviations, (2) the sample sizes for each of the distributions is only on the order of n = 10 so it cannot be assumed to be normally distributed, and (3) this test only compares pairs of variables; we had three groups over which to compare. We have instead employed the use of the non-parametric Kruskal-Wallis test for comparing the medians between distributions for multiple groups. After this test, we employed Tukey’s honest significant difference for post-hoc analysis to obtain the p-value for each pair. We have corrected the text to explain our statistical approach, with the amended text provided below.

We compared each parameter across conditions using the Kruskal-Wallis test; this test is non-parametric, does not assume normal distribution, and looks to determine if the distributions are significantly different based on the median. Post-hoc evaluation using Tukey honest significant difference was performed to obtain the p-value for each pair of tested conditions.

Furthermore, the p-values for each quantity reported in Table 3 and S2 Table have been updated accordingly. Subject data was also re-evaluated during this exercise, with no significant alterations to the statistical outcomes observed.

During this process, we identified an error in the units reported for Kinetic Energy, Flow Energy Loss, and Vortex Strength in Table 3. Originally intended to be presented in units of mJ/m, mW/m, and cm^2^/s, respectively, our reported values were area averaged, resulting in units of mJ/m^3^, mW/m^3^, and s^-1^. To maintain consistency with current literature, we have rectified this issue by removing the area averaging step.

Below I have some minor comments:

1) Line 185: There is a sentence that consists of "This." I presume that is a typo, please remove.

This was addressed in a response to Reviewer #3 above.

2) Line 205: the prhase "to under differences in filling", I presume you meant understand differences in filling

The text has been corrected accordingly.

3) Line 236: The authors say that SV is increased compared to control LV and RV in the prenatal setting but the statistics would suggest that SV in SRV is increased compared to the LV and trending in the RV (p=0.06)

We have corrected the text to properly reflect that while comparison to the control LV was significant, comparison to the control RV was trending toward significance. The amended text is provided below.

Major differences were observed for morphology and systolic parameters. SV (ml) and CO (ml/min) were elevated for the SRV compared to the control LV by a nearly two-fold statistically significant difference, and trending toward significance compared to the control RV (p = 0.060).

4) Line 275: I believe the reference was to the E/A ratio but in the sentence, it says E/, please fix this for clarity

This was in refence to E/A ratio; the text has been corrected for this change.

5) Line 287: "Stronger EL was observed both diastole", might want to add the during after observed

The text has been corrected accordingly.

6) Line 311: The median E/e' in SRV group during postnatal was listed as 1.57, which is a typo based on the table showing 15.7.

This has been addressed in above for a comment from Reviewer #3.
