# Supplementary material for: Fetal and neonatal echocardiographic analysis of biomechanical alterations for the systemic right ventricle heart
Source: PLoS One. 2024 Sep 19;19(9):e0308645. doi: 10.1371/journal.pone.0308645 (PMC11412552; doi:10.1371/journal.pone.0308645)
Supplement: Attachment — Submitted filename: Response for Reviewers.docx [file pone.0308645.s006.docx]

## Overview and response structure

We thank the reviewers for their valuable input throughout the review process. We have addressed their revisions to clarify the presented work further. Each point is addressed with a response and the corresponding excerpt from the revised final manuscript. Our response is provided in red Arial, and the text excerpt is in blue Arial.

## Response to reviewers

**Academic Editor:**

The authors are possibly trying to answer the wrong question or using the wrong comparisons to answer it.

We don’t really know what predestines some systemic RVs to fail while others do well, and I suspect that is what is behind a study like this where standard echo parameters are not sensitive enough... But I think this study may have within it the data which may start to unravel this...

The fetal systemic RV is doing much the same job as it is postnatally (in a 'single ventricle' circulation, whereas there are much larger changes in the loading on the systemic LV (or indeed the subpulmonary RV) in the biventricular circulation. Thus, using the normal hearts as controls is counterintuitive at least to me. To me the 'money' is in the changes from pre to postnatal parameters on the same ventricle, +/- the same ventricle in a biventricular circulation eg ccTGA, or indeed TGA pre repair. Comparing the CHANGES to the changes for the biventricular LV may also be of interest but there are much greater differences in the loading conditions in this especially depending on timing of ductal closure as well as the timing of the drop in PVR. which varies per individual.

So I think possibly reanalyzing these data in a different way would be of more physiologic interest. I really don’t think comparing absolute measurements of any of these parameters between different ventricular morphologies makes much sense as intrinsically they are not only constructed differently at the cellular and fibromuscular architecture level but they have different shapes, different loading conditions and even different interventricular interactions depending on the status of the other ventricle

So to me this study demonstrates some novel techniques but the questions as to their utility remain, to me as yet, completely unaddressed. Maybe I have missed the point but I don’t think this is publishable without some reference to this.

We appreciate the editor's comments and suggestions. The primary aim of this study is to develop and demonstrate tools and methodology that will enable identifying factors contributing to the success or failure of systemic right ventricles (SRVs) of the single ventricle subtype. There is a current lack of innovative technology for assessing ventricular function in congenital heart disease, particularly in SRV hearts. Our work focuses on developing and validating a methodology using a scalable platform applicable to preterm and neonatal patients. Unlike cardiac MRI, which poses workflow and safety challenges in neonates and has limited spatial and temporal resolution for small hearts, echocardiography offers a viable alternative. Our study employs innovative imaging analysis to shed light on the fetal-to-postnatal heart transition in normal and SRV hearts, promising new insights. In response to feedback, we have emphasized our findings' clinical relevance and limitations in the Discussion. We believe our rigorously conducted study introduces novel techniques that advance echocardiography. Moving forward, we will validate these findings in a larger cohort, including single ventricle and TGA patients, to demonstrate clinical applicability.

**Reviewer #5:**

The authors compared echocardiography-derived biomechanic data of 10 systemic right ventricles subjects with that of normal control subjects in both prenatal and postnatal period. and they showed increased stroke volume and cardiac output but also increase energy loss, reduced diastolic mechanics in those with systemic RV.

First, the authors work is novel and do demonstrate increased understanding of altered cardiac mechanics by using advance echocardiographic technique. This may help to increase our understanding of the pathophysiology.

However, the comparison between the patients and controls is compounded by the fact that the patients had unbalanced ventricles. The right ventricle in the subjects is not only pressured loaded when compared to normal subpulmonary ventricle, but also volumed loaded because the systemic RV has to provide driving force to both pulmonary and systemic circulation, which is unlike in other systemic RV (such as TGA or CCTGA) that the systemic RV only sustains systemic circulation.

As such their findings have to be interpreted with caution and could not be applied to TGA or CCTGA, both also having systemic RV before surgical switch operation. And this should be described and addressed in their discussion that they are studying a subset of systemic RV.

We acknowledge the point the reviewer has made. We have revised our Discussion to address this concern, noting that we are focusing on a subset of systemic RV. Moreover, we indicate this as need for future investigation in a study with more clinical focus. The revised text is provided below.

Our study cohort size (10 SRV subjects) may not yet capture the statistics of the broader population. Furthermore, the study observed fetuses at 33 weeks gestational age, so the biomechanics quantified may not represent those immediately before birth. Moreover, the cohort comprises a diverse population of single ventricle subtype SRV subjects exhibiting varying hemodynamics and volume loading conditions. Given this diversity, drawing definitive conclusions regarding altered biomechanics and hemodynamics during the perinatal transition necessitates investigation in cohorts with stringent exclusion criteria, focusing on individuals with more consistently similar findings. Additionally, the findings of this work cannot be applied to other SRV subtypes, such as transposition of the great arteries (TGA) or congenitally corrected TGA. These subtypes also have an SRV that sustains the systemic circulation before any surgical switch operation. These subtypes will also be considered in future studies when focusing on individuals with more consistently similar findings.

**Reviewer #6:**

This paper is well-structured, with detailed results, and a complete and comprehensive discussion, also acknowledging the study's limitations. The data presented are original and interesting, and the authors have clearly emphasized the lack of data in this field and the potential benefit and knowledge that their paper could provide.

Some paragraphs are challenging for some reader that has no strong familiarity with the algorithm beyond some of the measurements adopted. A more easy explanation of the novel hydrodynamic parameters would enhance the reader's understanding. Especially the image analysis workflow section [130-248] maybe should be simplified and further summarized with the help of more diagrams or Figures.

We recognize the challenges that some readers face in trying to understand the detailed explanation of the algorithms. We do believe the mathematical foundation provided is critical to this work and would add transparency, but it may alienate some audiences. We have moved most of our technical description to a supporting document (appendix) and made reference to the proper location for this detailed information as necessary in the text. The revised text describing the workflow is provided.

**Image analysis workflow**

The analysis workflow, summarized in Fig 1, outputs ventricular cardiac biomechanics measurements from B-mode and CFI ALAX recordings. These modalities are utilized because sonographers are well-trained in their recording, which enhances analysis consistency. The workflow automates measurements, enabling once challenging and highly user-variable analysis to become routine. All algorithms were run in MATLAB (The MathWorks, Natick, Massachusetts).

**Step 1: Tracking user input annulus and apex positions**

A single set of user inputs marking the ventricle apex and atrioventricular (AV) annulus positions on the initial recorded frame, depicted as the red and yellow positions in Fig 1-1, is required per scan. The input AV annulus positions are located at the annulus-septal wall and annulus-lateral wall junctions. These positions are tracked temporally using a speckle tracking algorithm based on the Fourier domain cross-correlation [18,19]. A more detailed description of the tracking algorithm is provided in the S1 Text.

The tracked positions provide measurements of ventricle relaxation that occurs during diastole and contraction that occurs during systole [20], driven by the AV annulus. Peak annulus velocities for systolic ejection (s’) and early diastolic filling (e’) are automatically measured from the velocity timeseries, also depicted in Fig 1-1, obtained through the tracking process using peak finding. Automated speckle tracking mitral annulus position and velocity measurements has been previously validated with adult patients [21].

**Step 2: Global longitudinal strain**

A novel algorithm is used to measure global longitudinal strain (GLS) from the whole ventricle image [22]. This algorithm overcomes limitations for strain estimation associated with manual tracings of the chambers [4,14] and the use of speckle pattern matching kernels [23,24] which are sensitive to image spatial and temporal resolution. B-mode recording frames are co-registered using tracked positions and the ventricle image is isolated, shown in Fig 1-2. For co-registered frame pairs containing the ventricle, a specialized logarithm-scaled kernel estimates global longitudinal strain rate (GLSr) between frames. The GLSr estimates are integrated to resolve GLS. Peak GLS $\left( \left| \mathrm{GLS} \right|,max \right)$ is output to quantify deformation from the GLS timeseries using peak finding. A more detailed description of the GLS algorithm is provided in the S1 Text.

**Step 3: Unsupervised chamber segmentation**

The unsupervised segmentation algorithm (ProID) automates ventricle detection and volume estimation [25]. The algorithm identifies ventricle boundaries using a machine vision algorithm [26] that finds the shortest path of pixels around the ventricle image, shown in Fig 1-3. ProID addresses contrast-to-noise and resolution issues common in natal imaging [14] with an echocardiogram-specific cost-matrix. The tracked positions from **Step 1** are used to initialize ProID for each frame. A more detailed description of the ProID algorithm is provided in the S1 Text.

Segmentation-derived volumes are computed from the identified boundaries using Simpson rule. Previous research demonstrated the accuracy of Simpson's rule in estimating actual volume using animal models in fetal echocardiography [27]. End-diastolic volume (EDV) was measured at the initial volume of each cycle in the processed volume timeseries. End-systolic volume (ESV) was determined using peak finding for the minimum volume in the cardiac cycle. Stroke volume (SV), calculated as the difference between EDV and ESV for each cycle, and cardiac output (CO), representing the stroke volume multiplied by heart rate, serve as output measures for systolic function.

**Step 4: Color Flow Imaging hemodynamics analysis of diastolic flow**

Doppler vector reconstruction (DoVeR) resolves the underlying 2D velocity vector field of blood flow in the ventricles from color Doppler imaging using the relationship between flow rate and fluid rotation [28]. DoVeR uses the tracked positions and ProID to segment the ventricle in each frame. These segmentations are used to set boundary conditions for DoVeR, shown in Fig 1-3. A more detailed description of the DoVeR algorithm is provided in S1 Text.

The DoVeR ventricular blood flow velocity vector fields, denoted as $\vec{u}$, are evaluated to quantify biomechanics parameters, including peak early filling velocities (E), kinetic energy (KE), energy loss (EL), and vortex strength (VS). Furthermore, the velocities are used to estimate relative pressure, from which the annulus-to-apex recovery pressure difference (recovery $\Delta P$) from the intraventricular pressure difference (IVPD) and AV valve center to minimum pressure distance (AV-to-P_min_) are computed.

The KE, a measure of the amount of energy blood flow has due to its motion, is calculated by integration of the summed square of the velocity measurements over the ventricle area,

$$KE=\int_{A} \frac{1}{2}\rho\sum\vec{u}^{2}dS.$$

*(1)*

Here, $\rho$ is the density of blood, assumed as 1030 ${kg}/{m^{3}}$. The EL, a measure of the KE lost due to rotation or acceleration/deceleration, is calculated by integration of the summed spatial gradients of the velocity measurement over the ventricle area,

$$EL=\int_{A} \frac{1}{2}\mu\sum_{ij} \left( \frac{\partial u_{i}}{\partial x_{j}}+\frac{\partial u_{j}}{\partial x_{i}} \right)^{2}dS.$$

*(2)*

Here, $\mu$ is the blood viscosity which is assumed as $3 mPa\cdot s$, and *i,j* are indices representing the spatial components of the velocity vector field in the horizontal and vertical directions. The VS, an absolute quantity of the total amount of rotation observed in the flow field, is measured from vorticity $\left( \vec{\omega} \right)$, the potential for blood flow rotation, integrated over the ventricle area. Vorticity is computed taking the curl of the velocity field $\vec{u}$,

$\vec{\omega}=\nabla\times\vec{u}$.

*(3)*

Thus, VS is computed as,

$$VS=\int_{A} \left| \vec{\omega} \right|\cdot dS.$$

*(4)*

Relative pressure of the DoVeR blood flow velocity vector fields, is estimated by integration of the pressure gradients [13] which are computed based on the Navier Stokes Equation (NSE),

$$\frac{\partial P}{\partial x_{i}}=-\rho\left( \frac{\partial u_{i}}{\partial t}+u_{j}\frac{\partial u_{i}}{\partial x_{j}} \right)+\mu\frac{\partial^{2}u_{i}}{\partial x_{j}\partial x_{j}}.$$

*(5)*

The IVPD is calculated as the difference between pressure at the AV (P_TV_) and the ventricle (P_Apex_) such that,

$$IVPD=\Delta P=P_{TV}-P_{Apex}.$$

Equation 7.

The E is measured by peak finding from the velocity timeseries for each cardiac cycle. Recovery $\Delta P$ is measured as the minimum IVPD measurement across each cardiac cycle. The AV-to-P_min_ is measured as the distance between minimum and maximum pressure measurements for each timepoint across each cardiac cycle. Dimensionless quantities for E/e' and E/A, with peak late filling velocity (A) measured by peak finding along the velocity timeseries, were also computed. Only diastolic flow assessment was emphasized, as the traditional 4-chamber apical view does not adequately profile the outflow in the imaging plane of the transducer. Advanced hydrodynamics parameters (i.e. KE, EL, VS) were obtained by averaging across the diastolic period of each cardiac cycle for their respective timeseries.

The results are well presented with appropriate use of tables and Figures to illustrate key findings. Maybe the clinical relevance of the findings should be further stressed. The potential clinical applications of the novel parameters are generic and should be better articulated in the conclusion. Highlighting the clinical implications of these findings more explicitly would strengthen the impact of the results.

We recognize the reviewer’s point that clinical relevance should be further stressed. While this work is intended to demonstrate the tools with less focus on clinical insight, we have provided additional context at the end of our Discussion section. The revised text is provided below.

In SRV patients with single ventricle subtypes, such as hypoplastic left heart syndrome, the RV rapidly enters heart failure within the first week of life due to pulmonary over-circulation and systemic under-circulation. Treatments like Prostaglandin 1 (PGE 1) are administered to counter these effects, but patients are often quickly moved to palliative interventions (e.g., Norwood procedure or hybrid palliation). Patient progression to heart failure is inconsistent, suggesting that the underlying mechanisms are not fully understood. The early period treatments and decisions are crucial due to rapid physiological changes, which are often not reliably captured by current medical imaging analysis. The methods described here could provide improved sensitivity to these physiological changes, offering a better understanding of ventricular work and optimizing patient management.

In summary, the paper is well-written and provides valuable datas. With minor revisions, it would be a fit for publication.
